# The Magnesium Concentration in Yeast Extracts Is a Major Determinant Affecting Ethanol Fermentation Performance of *Zymomonas mobilis*

**DOI:** 10.3389/fbioe.2020.00957

**Published:** 2020-08-31

**Authors:** Runxia Li, Mingjie Jin, Jun Du, Mian Li, Shouwen Chen, Shihui Yang

**Affiliations:** ^1^State Key Laboratory of Biocatalysis and Enzyme Engineering, Environmental Microbial Technology Center of Hubei Province, School of Life Sciences, Hubei University, Wuhan, China; ^2^School of Environmental and Biological Engineering, Nanjing University of Science and Technology, Nanjing, China; ^3^China Biotech Fermentation Industry Association, Beijing, China; ^4^Zhejiang Huakang Pharmaceutical Co., Ltd., Quzhou, China

**Keywords:** *Zymomonas mobilis*, ethanol fermentation, nitrogen sources, yeast extract, magnesium, RNA-Seq, stress responses

## Abstract

*Zymomonas mobilis* is a model ethanologenic bacterium for diverse biochemical production. Rich medium (RM) is a complex medium that is routinely used to cultivate *Z. mobilis*, which contains carbon sources such as glucose, nitrogen sources such as yeast extract (YE), and KH_2_PO_4_. Glucose consumption and cell growth of *Z. mobilis* is usually coupled during ethanol fermentation. However, sometimes glucose was not consumed during the exponential growth phase, and it took extended time for cells to consume glucose and produce ethanol, which eventually reduced the ethanol productivity. In this study, the effects of different nitrogen sources, as well as the supplementation of an additional nitrogen source into RM and minimal medium (MM), on cell growth and glucose consumption of *Z. mobilis* were investigated to understand the uncoupled cell growth and glucose consumption. Our results indicated that nitrogen sources such as YE from different companies affected cell growth, glucose utilization, and ethanol production. We also quantified the concentrations of major ion elements in different nitrogen sources using the quantitative analytic approach of Inductively Coupled Plasma Optical Emission Spectroscopy (ICP-OES), and demonstrated that magnesium ion in the media affected cell growth, glucose consumption, and ethanol production. The effect of magnesium on gene expression was further investigated using RNA-Seq transcriptomics. Our results indicated that the lack of Mg^2+^ triggered stress responses, and the expression of genes involved in energy metabolism was reduced. Our work thus demonstrated that Mg^2+^concentration in nitrogen sources is essential for vigorous cell growth and ethanol fermentation, and the difference of Mg^2+^concentration in different YE is one of the major factors affecting the coupled cell growth, glucose consumption and ethanol fermentation in *Z. mobilis.* We also revealed that genes responsive for Mg^2+^ deficiency in the medium were majorly related to stress responses and energy conservation. The importance of magnesium on cell growth and ethanol fermentation suggests that metal ions should become one of the parameters for monitoring the quality of commercial nitrogen sources and optimizing microbial culture medium.

## Introduction

The Gram-negative bacterium *Zymomonas mobilis* is a natural ethanologen with many desirable industrial biocatalyst characteristics, which include high glucose consumption rate, high specific ethanol productivity, high ethanol tolerance, a broad pH range for production (pH 3.5–7.5), and its status being generally regarded as safe (GRAS) (Rogers et al., 1984, 2007; Panesar et al., 2006; He et al., 2014; Yang et al., 2016a; Wang et al., 2018). *Z. mobilis* utilizes glucose for ethanol production faster than *Saccharomyces cerevisiae* because of its high cell surface area which leads to higher ethanol productivity (Conway, 1992; Lawford and Rousseau, 1997; Rogers et al., 2007; He et al., 2014; Yang et al., 2016a; Wang et al., 2018).

Additionally, Compared with *S. cerevisiae*, the preferred biocatalyst using the Embden-Meyerhof-Parnas (EMP) pathway for industrial ethanol production with mature infrastructures, *Z. mobilis* has improved ethanol yield by using the Entner-Doudoroff (ED) pathway with 50% less ATP produced relative to the EMP pathway (Conway, 1992; Kingston et al., 1996; Dien et al., 2003). Recently, Jacobson et al. (2019) established a network-level approach that integrates quantitative metabolomics with ^2^H and ^13^C metabolic flux analysis to investigate the *in vivo* thermodynamics of the ED pathway and central carbon metabolism in *Z. mobilis*. Their result is consistent with previous *in silico* prediction (Flamholz et al., 2013) and demonstrated that ED pathway is twice as thermodynamically favorable as the EMP pathway in *E. coli* or *S. cerevisiae* (Jacobson et al., 2019).

Nitrogen sources in the medium are reported to affect the growth of *Z. mobilis.* For example, the morphology of *Z. mobilis* CP3 changes when cultured in a medium with a low nitrogen source (Ju et al., 1983). A medium commonly used to culture *Z. mobilis* is rich medium (RM). RM contains carbon sources such as glucose, nitrogen sources such as yeast extract (YE), and KH_2_PO_4_ that is often used as the phosphorus and potassium source and a buffering agent. Although nitrogen sources like peptone, corn steep liquid, and even N_2_ can be used to sustain the cell growth of *Z. mobilis* (Ju et al., 1983; Lawford and Rousseau, 1997; Kremer et al., 2015), YE is the preferred nitrogen source because it can provide nitrogenous compounds, carbon, sulfur, trace nutrients, vitamin B complex and other important growth factors for various microorganisms (Zarei et al., 2016).

Despite the fact that manufacturers produce different YE to have the same composition of total nitrogen content and free amino acid nitrogen, YE produced by different companies has different trace components such as growth factors and metal ions due to the differences in their production processes. This then affects the microbial cell growth because vitamins and metal ions (e.g., Mg^2+^, Cu^2+^, Zn^2+^, and Fe^2+^) are cofactors of enzymes involved in various metabolic activities. For example, Kosaka et al. (2020) investigated the effect of different metal ions on the thermotolerance of *Z. mobilis* TISTR548 and related mutants, and demonstrated that the addition of Mg^2+^ and K^+^ reduced intracellular reactive oxygen species (ROS) accumulation at critical high temperature (CHT) with an increase of CHT by 1°C, which is probably due to the stabilization of both outer and inner membranes as well as the maintenance of homeostasis for cellular metabolism by the addition of Mg^2+^ and K^+^. In addition, the effect of supplementation of zinc on the ethanol fermentation performance of the self-flocculating yeast in the continuous ethanol fermentation was studied (Zhao et al., 2009; Xue et al., 2010; Ismail et al., 2014), and the roles of zinc and zinc containing proteins in yeast metabolism and cellular stress responses were summarized (Zhao and Bai, 2012).

With all nutrients needed for cell growth in the culture medium including a nitrogen source from YE, cell growth of *Z. mobilis* ZM4 is generally expected to be coupled with its glucose consumption and ethanol production. However, results and literature reports in a few studies from different research groups indicated that cell growth of *Z. mobilis* ZM4 and its glucose consumption were uncoupled, glucose was not completely consumed for ethanol production when cells reached stationary phase (Jones and Doelle, 1991; He et al., 2012; Yang et al., 2014a; Xia et al., 2018; Duan et al., 2019). This uncoupling phenomenon between growth and fermentation performance has also been reported in other microorganisms, including yeast, resulting in a prolonged fermentation time and decreased ethanol productivity (Cot et al., 2007; Pagliardini et al., 2013). For example, ethanol production of yeast cells was reported to be related to the length of the uncoupling phase during the batch fermentation process (Pagliardini et al., 2013).

A previous study in *E. coli* demonstrated that the inadequate amount of magnesium in the rich medium of buffered tryptone broth supplemented with glucose (TB7-glucose) is the major determinant leading to the uncoupled cell growth and glucose consumption (Christensen et al., 2017). They further demonstrated that the supplementation of sufficient magnesium can increase cell growth yield in other peptide-based media when carbon is in excess. In addition, their study also showed that magnesium can increase cell growth of multiple *E. coli* strains and other bacterial species such as *Bacillus subtilis* and *Vibrio fischeri* (Christensen et al., 2017). Recently, Lozano Terol et al. (2019) studied protein overexpression for *E. coli* BL21 and five derived mutants in M9 minimal medium and TB7 complex medium, and discovered a similar phenomenon that carbon source of glucose or glycerol was not consumed during the exponential growth phase in TB7 complex medium but not M9.

The uncoupling of growth and fermentation in the late growth phase could be beneficial for ethanol fermentation since carbon source will be channeled into ethanol production instead of biomass buildup especially for yeast ethanol production. However, the uncoupling of growth and glucose consumption at early growth phase is unfavorable since there will be no sufficient cell biomass for efficient sugar fermentation to ethanol, which is mostly due to stressful physical and chemical growth conditions, such as extreme temperature, extreme pH, and toxic compounds in the medium. Limited nutrient sources of carbon, nitrogen and metal ions used for fermentation were also reported to affect cell growth and fermentation performance (Jones and Doelle, 1991; Cot et al., 2007; Pagliardini et al., 2013; Yang Y. et al., 2018). Although the effect of diverse nitrogen sources on cell growth and fermentation performance has been reported in various microorganisms, such as the ethanologenic yeast *S. cerevisiae* and lactate-producer *Sporolactobacillus inulinus* (Martinez-Moreno et al., 2012; Kemsawasd et al., 2015; Klotz et al., 2017), metal ions within nitrogen sources and their impact on cell growth and fermentation have not been investigated extensively.

To understand the uncoupling phenomenon of cell growth and glucose consumption for both efficient carbon utilization and maximum ethanol productivity, we investigated the effects of different nitrogen sources on cell growth, glucose consumption and ethanol production of *Z. mobilis* in this study to help achieve the goal of optimal titer, rate, and yield for economic bioethanol production using *Z. mobilis*. Our results demonstrated that glucose consumption and ethanol production can be coupled with cell growth by changing the nitrogen sources in the rich medium, and the concentration of metal ions such as Mg^2+^ in the nitrogen source is a major factor affecting cell growth and ethanol production.

## Results and Discussion

### Effect of Supplementation of Nitrogen Sources Into RM

To understand the uncoupling of cell growth, glucose consumption and ethanol production in *Z. mobilis*, we evaluated factors in the medium affecting cell growth. The recipe of RM for *Z. mobilis* is relatively simple with carbon sources such as glucose, phosphate sources of KH_2_PO_4_, and YE as the source of nitrogen, minerals, and vitamins. Thus, 10 mM NH_4_Cl was added into RM using YE from the company OXOID (RM^OXOID^) to increase inorganic nitrogen content. However, extra NH_4_Cl supplemented into RM^OXOID^ did not reduce the time for glucose utilization and ethanol production of ZM4 (data not shown).

**FIGURE 1:**
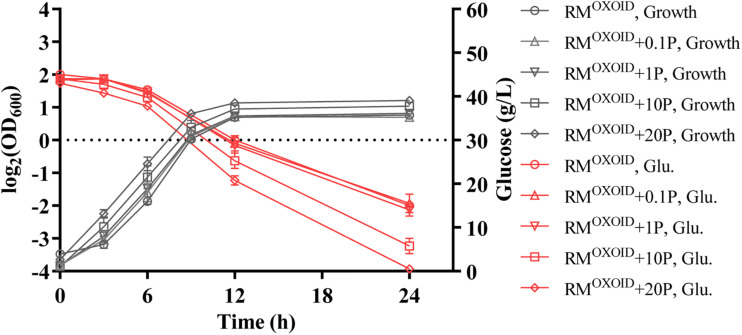
Cell growth and glucose (Glu) consumption of ZM4 in rich medium (RM) using yeast extract from OXOID company (RM^OXOID^) with extra peptone (P) supplemented at concentrations of 0.1, 1, 10, and 20 g/L.

The effect of supplementing different organic nitrogen sources such as peptone and tryptone were then tested. Our results showed that the supplementation of peptone did not reduce the time of glucose utilization. It still took more than 24 h for cells to consume all glucose in the media with 20 g/L peptone supplemented into RM^OXOID^. The final biomass in terms of OD_600_ value did increase slightly in correspondence to the increase of peptone added (Figure 1).

**FIGURE 2:**
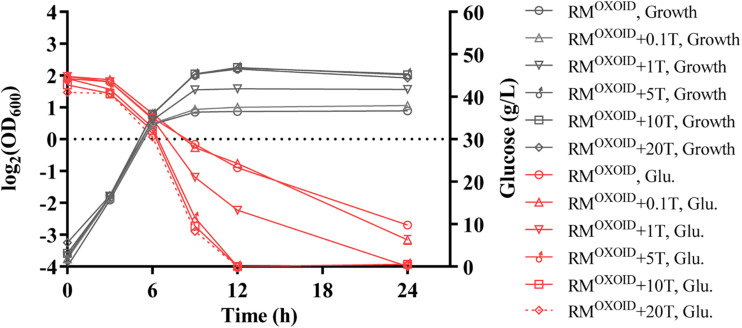
Cell growth and glucose (Glu) consumption of ZM4 in rich medium (RM) using yeast extract from OXOID company (RM^OXOID^) with tryptone (T) supplemented at concentrations of 0.1, 1, 5, 10, and 20 g/L.

Subsequently, different concentrations of tryptone were added in RM^OXOID^ in a separate experiment. The results showed that the addition of extra tryptone could significantly increase both cell biomass and glucose consumption of ZM4 (Figure 2). The addition of more than 5 g/L tryptone in RM^OXOID^ not only enhanced both cell growth and glucose utilization of ZM4, but also coupled the growth and ethanol fermentation of ZM4. The final highest OD_600_ value of ZM4 in RM^OXOID^ with 5 g/L tryptone increased from 1.8 to 4.7, and all glucose was completely consumed within 12 h. *Z. mobilis* in medium without tryptone only utilized half the glucose at the same time point of 12-h post-inoculation (Figure 2).

The positive effect of adding tryptone into RM^OXOID^ was further evaluated by comparing cell growth along with glucose utilization and ethanol production of ZM4 in both RM^OXOID^ and RM^OXOID^ + 5T at different temperatures of 30, 36, and 40°C. The results demonstrated that the supplementation of tryptone increased the growth and fermentation performance of ZM4 at each of these different temperatures, including the specific growth rate (**μ**), ethanol yield (***Y*_*p/s*_**), and productivity (***Q*_*P*_**) (Table 1).

**TABLE 1:** Glucose consumption (Y_*s*_), ethanol yield (*Y*_*p/s*_), theoretical ethanol yield (*%Y_*p/s*_*), ethanol productivity (*Q*_*P*_), and specific growth rate (μμ) of ZM4 in RM^OXOID^ and RM^OXOID^ supplemented with 5 g/L tryptone at different temperatures within 24 h.

**Conditions**	***Y*_*s*_ (g/L)**	***Y*_*p/s*_ (g/g)**	*%Y_*p/s*_* (g/g*100)	***Q*_*p*_ (g/L/h)**	**μ (h^–1^)**
30°C, RM^OXOID^	41.69 ± 0.27	0.39 ± 0.02	76 ± 4	0.67 ± 0.03	0.45 ± 0.01
36°C, RM^OXOID^	39.36 ± 0.25**	0.36 ± 0.02	70 ± 4	0.59 ± 0.04	0.45 ± 0.01
40°C, RM^OXOID^	39.40 ± 0.26**	0.37 ± 0.02	73 ± 4	0.61 ± 0.03	0.42 ± 0.00*
30°C, RM^OXOID^ + 5T	46.28 ± 0.04**	0.45 ± 0.00*	89 ± 1	1.75 ± 0.01**	0.49 ± 0.01*
36°C, RM^OXOID^ + 5T	46.29 ± 0.00**	0.43 ± 0.04	83 ± 8	2.19 ± 0.22*	0.55 ± 0.01**
40°C, RM^OXOID^ + 5T	46.08 ± 0.36**	0.44 ± 0.02	85 ± 4	2.23 ± 0.11**	0.53 ± 0.01*

Multiple comparisons were conducted using the values when cells were grown in RM^*OXOID*^ at 30°C as the control, *represents a significant difference (0.01 < P < 0.05), **represents a very significant difference (P < 0.01).

**FIGURE 3:**
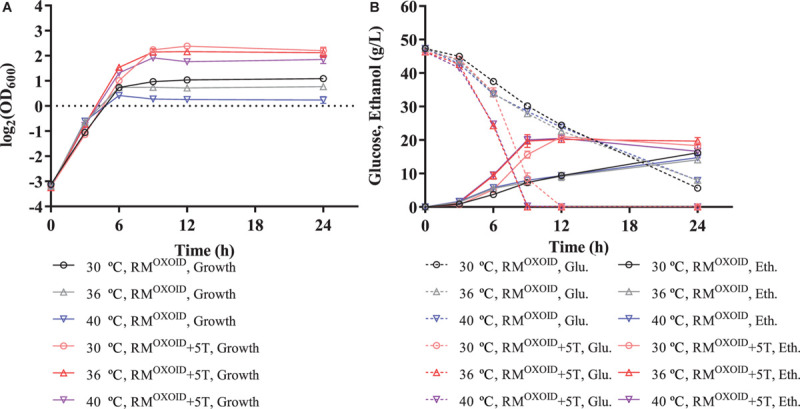
Cell growth **(A)**, glucose (Glu) consumption and ethanol (Eth) production **(B)** of ZM4 in rich medium (RM) using yeast extract from OXOID company (RMOXOID) at different temperatures of 30, 36, and 40°C with extra 5 g/L tryptone added.

It also increased cell growth, glucose consumption rate, and ethanol productivity of ZM4 at higher temperatures of 36 and 40°C than those at the normal temperature of 30°C in RM^OXOID^ supplemented with 5 g/L tryptone, although ethanol yields were similar among these conditions (Table 1 and Figure 3). Compared with a normal temperature of 30°C, the time that ZM4 utilized all glucose at 36 and 40°C in RM^OXOID^ with extra tryptone was reduced by about 3 h, but 1 day was not sufficient for ZM4 to completely consume glucose at any of the aforementioned temperatures in RM^OXOID^ (Figure 3). ZM4 took less time to completely utilize glucose with maximum ethanol production achieved in RM^OXOID^ + 5T at 36 and 40°C (9 h) than at 30°C (12 h) with a corresponding higher growth rate and ethanol productivity, which was increased from 1.75 ± 0.01 at 30°C to 2.19 ± 0.22 and 2.23 ± 0.11 at 36 and 40°C, respectively (Table 1 and Figure 3).

### Effect of Exchanging YE From OXOID With Different Brand Ones in RM

As briefly mentioned above, YE distributed by different companies are produced with different processes which may lead to different amounts of total nitrogen and trace elements such as growth factors and metal ions. Besides adding extra nitrogen sources into the RM^OXOID^ as discussed above (Figures 1–3), we also tested the effect of YE from different companies, including those from Becton Dickinson (YE^BD^) and Sangon Biotech Co., Ltd. (YE^SG^, Shanghai, China). In addition, another industrial nitrogen source of corn steep liquid (CSL) from Macklin was evaluated at the same time as the two yeast-based nitrogen sources discussed above. All media used in this research are listed in Tables 5, 6.

**FIGURE 4:**
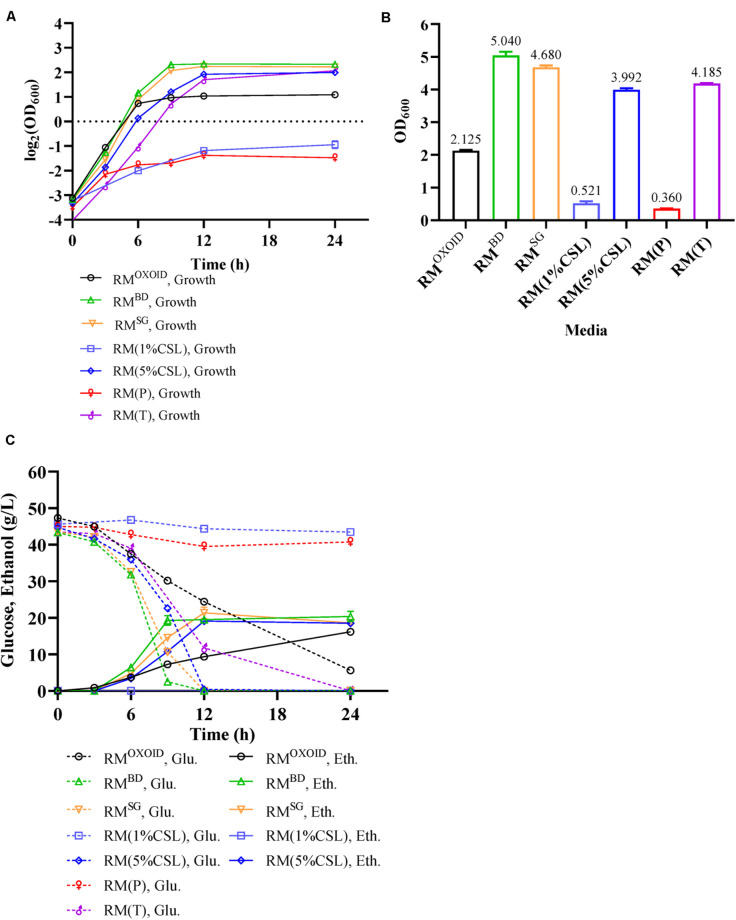
Cell growth **(A)**, final OD_600_ value **(B)**, as well as glucose (Glu) consumption and ethanol (Eth) production **(C)** of ZM4 in RM with different nitrogen sources.

YE^OXOID^ in RM^OXOID^ was replaced with different nitrogen sources including peptone (P), tryptone (T), CSL, YE^BD^, and YE^SG^. Cell growth, glucose utilization, and ethanol production of ZM4 cultured in these media were then examined. Our results showed ZM4 performed differently in these media (Figure 4). The biomass of ZM4 in RM(T) was about two times higher than that in RM^OXOID^, while the biomass of ZM4 in RM(P) was the lowest (Figure 4B). Tryptone appeared to be better than YE^OXOID^ and peptone for cell growth, which was consistent with the aforementioned experiments of supplementation of nitrogen source into RM^OXOID^ (Figures 1, 2).

Except for the fact that the specific growth rates of ZM4 in RM with 1% of peptone or CSL as the sole nitrogen source were lower, which can be seen from the slopes of the lines in the exponential phase, the specific growth rates of ZM4 in other RM were almost the same (Figure 4A). Compared with YE^OXOID^ as the sole nitrogen source, YE^BD^, YE^SG^, and 5% CSL all increased the final biomass of ZM4 (Figure 4B), reduced the time of glucose consumption (Figure 4C), and coupled cell growth and ethanol production. The biomass of ZM4 in RM^BD^ was more than two times of that in RM^OXOID^ and the time for glucose consumption in RM^BD^ was about two-thirds shorter than that in RM^OXOID^ (Figures 4B,C). YE^SG^, though cheaper than YE^BD^, was almost just as good. In the future, YE^SG^ could be used in large-scale fermentation for ethanol production, and the appropriate nitrogen source can be chosen as needed based on the results above (Figures 1–4).

### Effect of Exchanging (NH_4_)_2_SO_4_ in MM With Different Organic Nitrogen Sources

Although the RM recipe is relatively simple containing only three ingredients as discussed above, the composition of organic nitrogen sources such as YE is still complicated containing nitrogen, vitamins, metal ions, etc. Because the only nitrogen source in minimal medium (MM) is the inorganic nitrogen source (NH_4_)_2_SO_4_, it was selected to further examine the effect of different organic nitrogen sources on ZM4. The representative organic nitrogen sources used to replace (NH_4_)_2_SO_4_ in MM included YE^BD^, YE^OXOID^, and 5%CSL.

**FIGURE 5:**
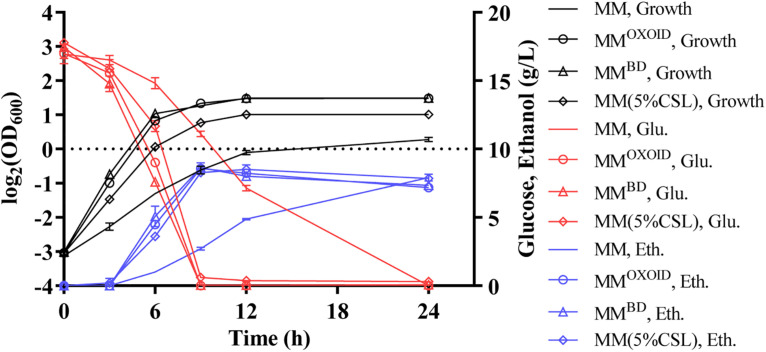
Cell growth, glucose (Glu) consumption and ethanol (Eth) production of ZM4 in MM containing 20 g/L glucose with different nitrogen sources.

Our results demonstrated that the replacement of inorganic nitrogen source (NH_4_)_2_SO_4_ with organic nitrogen source enhanced cell growth, glucose utilization, and ethanol productivity (Figure 5). Interestingly, ZM4 had similar growth and glucose consumption rates in two MM media with either YE^BD^ or YE^OXOID^ as the sole nitrogen source (Figure 5). These results differed from those when YE^BD^ or YE^OXOID^ was used as the sole nitrogen source in RM where ZM4 grew better in RM with YE^BD^ than in RM with YE^OXOID^ as the nitrogen source (Figure 4). Therefore, some ingredients must exist in MM which RM lacks when YE^OXOID^ is used as the nitrogen source. This difference is contingent on compositions of NaCl, Na_2_MoO_4_, and MgSO_4_ which differ in the ingredients of the two media (Tables 5, 6).

**FIGURE 6:**
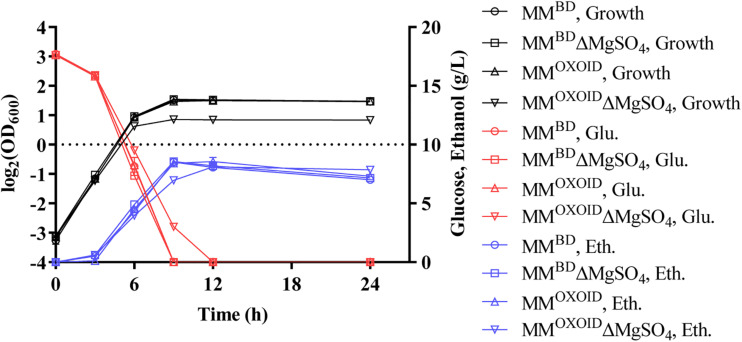
Cell growth, glucose (Glu) consumption and ethanol (Eth) production of ZM4 in MM containing 20 g/L glucose with original inorganic nitrogen sources replaced by YE^BD^ and YE^OXOID^, respectively, as well as in above two media with Mg^2+^ removed.

Since magnesium ion is a cofactor of diverse enzymes involved in various metabolic activities, we investigated the effect of removing the magnesium ion from both MM^BD^ and MM^OXOID^ to test the growth and fermentation performance of ZM4. The lack of Mg^2+^ in MM^BD^ had no effect on the growth of ZM4, but the biomass decreased in MM^OXOID^ lacking Mg^2+^ (Figure 6). This result indicates that the concentration of Mg^2+^ in YE^OXOID^ is different from that in YE^BD^, and Mg^2+^ plays important roles in cell growth of *Z. mobilis*, and Mg^2+^ was likely one of the factors affecting the coupling of cell growth, glucose consumption and ethanol production.

### Determination of Concentrations of Ions in Different Nitrogen Sources and the Impact of the Mg^2+^ on Fermentation Performance

In order to verify this hypothesis, the concentrations of several major ion elements in different organic nitrogen sources were then measured by ICP-OES. The result showed that the concentrations of these ion elements (Fe, K, Mg, P, S) were different among different nitrogen sources with peptone and tryptone containing fewer of these ions (Table 2). This may be one reason why cell growth and fermentation performance of *Z. mobilis* in media using tryptone and peptone as the sole nitrogen source was not as efficient as in other media using YE (Figure 4).

**TABLE 2:** Concentrations of different ion elements in different nitrogen sources measured by ICP-OES.

**Nitrogen sources**	**Fe (mg/L)**	**K (mg/L)**	**Mg (mg/L)**	**Na (mg/L)**	**P (mg/L)**	**S (mg/L)**
1% YE^OXOID^	0.51	492.74	2.32 ± 0.72	19.16	159.72	58.69
1% YE^BD^	0.42	456.13	10.62 ± 0.64**	48.83	129.1	85.92
1% YE^SG^	0.28	311.83	14.49 ± 5.60	131.3	278.3	65.59
1% Peptone	0.13	5.52	0.11 ± 0.07	27.8	2.67	5.21
1% Tryptone	0.13	1.07	6.87 ± 5.87	49.56	16.65	17.04
1% CSL	0.48	280.71	13.13 ± 4.82	20.61	198.13	56.88

Magnesium ion was tested twice while other ions were tested only once. Multiple comparisons of magnesium concentrations in different nitrogen sources were conducted with 1% YE^*OXOID*^ as a control, **represents a significant difference (P < 0.01).

At the same concentration of nitrogen sources, the Mg^2+^ in the YE from OXOID was 8.3 mg/L less than that from Becton Dickinson. Mg^2+^ is the component with the major difference between these two nitrogen sources of YE from OXOID and BD companies, and therefore could be the reason that cell growth and ethanol fermentation of *Z. mobilis* in RM^OXOID^ was not as efficient as in RM^BD^ (Table 2). Since 0.5 g/L MgSO_4_⋅7H_2_O containing 49 mg/L Mg^2+^ was provided in MM which was sufficient for cell growth (Tables 2, 4), the growth difference of *Z. mobilis* in MM^OXOID^ and MM^BD^ was therefore not as obvious as that in RM^OXOID^ and RM^BD^ (Figures 4, 5). The concentration of Mg^2+^ in peptone was the lowest, which could also be one reason that ZM4 grew poorly in RM(P) compared to other nitrogen sources (Figure 4). These results further confirm the importance of sufficient Mg^2+^ concentration in media for optimal cell growth, glucose consumption and ethanol fermentation. The result that the concentration of Mg^2+^ in tryptone was higher than that in YE^OXOID^ was consistent with the results of the nitrogen source supplement experiment (Kosaka et al., 2020), and provided a conjecture that perhaps the concentration of magnesium ion in nitrogen sources plays an important role in promoting temperature tolerance and cell growth (Figures 2, 3).

**FIGURE 7:**
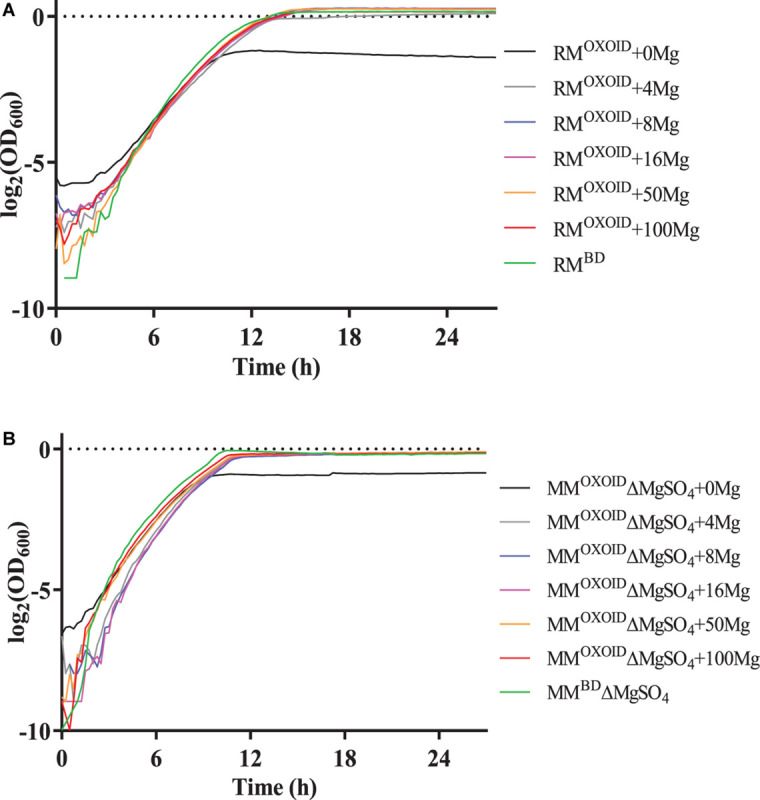
Cell growth of ZM4 in RM^OXOID^ **(A)** or MM^OXOID^ without MgSO_4_ (MM^OXOID^ΔMgSO_4_) **(B)** with Mg^2+^ supplemented at different concentrations of 0, 4, 8, 16, 50, and 100 mg/L. RM^BD^ and MM^BD^ΔMgSO_4_ were included as controls.

To verify the impact of Mg^2+^ on cell growth, different concentrations of Mg^2+^ were then added into the RM^OXOID^ and MM^OXOID^ without MgSO_4_ (MM^OXOID^ΔMgSO_4_), and the growth of ZM4 in these media was measured by the Bioscreen C (Figure 7). Our results indicated that in both RM^OXOID^ and MM^OXOID^ΔMgSO_4_, even a small amount of Mg^2+^ at 4 mg/L could boost cell growth significantly.

**FIGURE 8:**
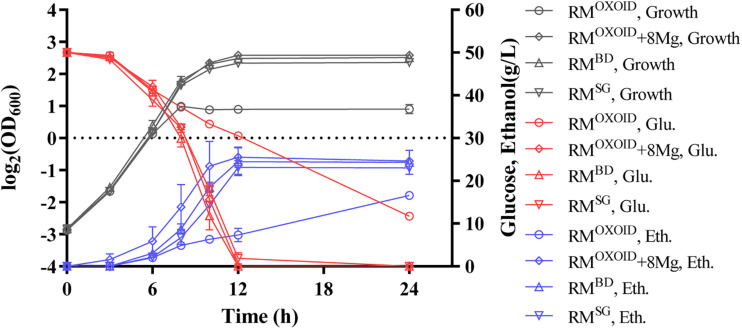
Cell growth, glucose (Glu) consumption, and ethanol (Eth) production of ZM4 in RM^OXOID^, RM^OXOID^ + 8Mg, RM^BD^, and RM^SG^. At least two independent experiments were carried out with similar results. Values are the mean of one representative experiment with two or more technical replicates. Error bars represent standard deviations.

Cell growth, glucose utilization, and ethanol production were further investigated using the shake flask experiment with OD_600_ values, glucose and ethanol concentrations measured (Figure 8). Consistent with the result of Bioscreen C (Figure 7), the supplementation of at least 8 mg/L of Mg^2+^ in RM^OXOID^ or MM^OXOID^ΔMgSO_4_ allowed ZM4 to grow as well as in RM^BD^ or MM^BD^ΔMgSO_4_. At the same time, cell growth, glucose consumption, and ethanol production of ZM4 in RM^OXOID^ with 8 mg/L Mg^2+^ added (RM^OXOID^ + 8Mg) were coupled as in RM^BD^ (Figure 8). This suggests that Mg^2+^ is crucial for cell growth, glucose consumption and fermentation performance of *Z. mobilis*, and a minimum concentration of at least 8 mg/L is needed for optimal cell growth and ethanol fermentation.

### Effects of Mg^2+^ on Gene Expression of *Z. mobilis*

After confirmation of the impact of Mg^2+^ on cell growth and ethanol fermentation of *Z. mobilis* (Figure 8), next-generation sequencing (NGS)-based RNA-Seq transcriptomics was further applied to identify genes responsive for the uncoupling of cell growth, glucose consumption and ethanol fermentation due to the difference of Mg^2+^ concentration in the medium.

**FIGURE 9:**
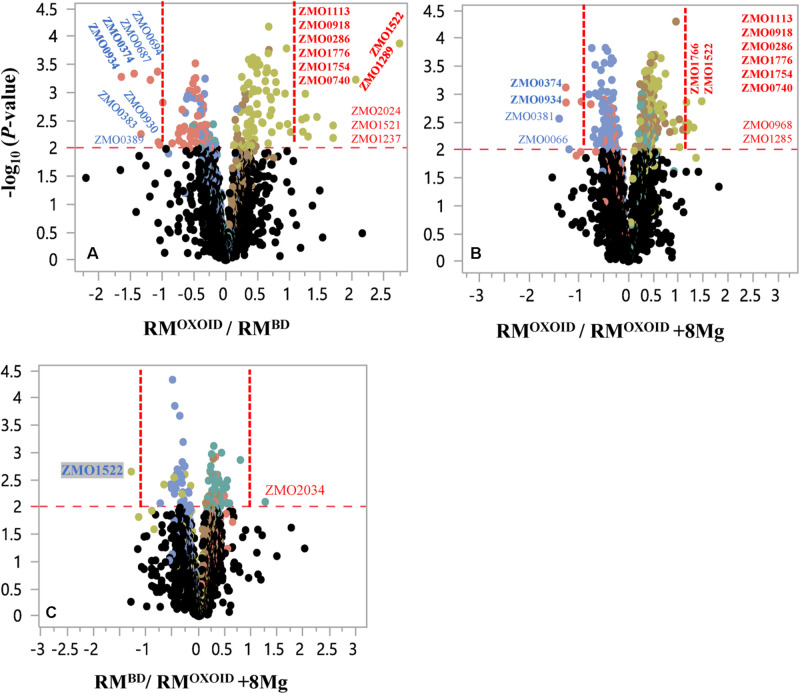
Volcano plots of significantly differentially expressed genes of *Z. mobilis* cultured in RM^OXOID^ and RM^BD^ (RM^OXOID^/RM^BD^, **(A)**, RM^OXOID^ and RM^OXOID^ + 8Mg (RM^OXOID^/RM^OXOID^ + 8Mg, **(B)**, as well as RM^BD^ and RM^OXOID^ + 8Mg (RM^BD^/RM^OXOID^ + 8Mg, **(C)**. X-axis is the log_2_-based ratios between two conditions examined, and Y-axis is the -log_10_(*P*-value) of the difference. The dots above the horizontal red dash line indicate genes significantly differentially expressed, and the vertical red dash line indicate genes significantly differentially expressed with ratio greater than 2 (log_2_-based ratio greater than 1). Gene name with red and blue color font indicates up-regulated and down-regulated ones, respectively. Gene names with bold font indicate common ones between different comparisons of RM^OXOID^/RM^BD^, RM^OXOID^/RM^OXOID^ + 8Mg, and RM^BD^/RM^OXOID^ + 8Mg.

Despite that cell growth, glucose consumption and ethanol production exhibited apparent differences (Figure 8), RNA-Seq results showed that only a few genes were differentially expressed. There were seven and four genes down-regulated when *Z. mobilis* was cultured in RM^OXOID^ and RM^BD^ (RM^OXOID^/RM^BD^, Figure 9A and Table 3) as well as cultured in RM^OXOID^ and RM^OXOID^ + 8Mg (RM^OXOID^/RM^OXOID^ + 8Mg, Figure 9B and Table 4), respectively. Although genes up-regulated in RM^OXOID^ were more than those down-regulated (Figure 9 and Tables 3, 4), there were only 11 and 10 up-regulated genes identified when *Z. mobilis* was cultured in RM^OXOID^ and RM^BD^ (RM^OXOID^/RM^BD^, Figure 9A and Table 3) as well as cultured in RM^OXOID^ and RM^OXOID^ + 8Mg (RM^OXOID^/RM^OXOID^ + 8Mg, Figure 9B and Table 4), respectively. Two and eight of these down-regulated and up-regulated ones are common between these two comparisons (Figure 9 and Tables 3, 4).

**TABLE 3:** List of significantly differentially expressed genes between ZM4 cultured in RM^BD^ and RM^OXOID^.

**Name**	**Product**	**Ratio**	**−log_10_ (*P*-value)**
**Up-regulated gene in ZM4 cultured in RM^OXOID^ compared with RM^BD^**			
*ZMO1113*	NADH dehydrogenase	1.03	2.29
*ZMO0918*	Catalase	1.21	2.52
*ZMO0286*	DUF541 domain-containing protein	1.22	2.30
*ZMO1776*	Aminopeptidase N	1.26	2.98
*ZMO1754*	Succinate-semialdehyde dehydrogenase SSADH	1.27	2.55
*ZMO2024*	Hypothetical protein	1.30	2.21
*ZMO0740*	General stress protein CsbD	1.44	2.56
*ZMO1521*	Hypothetical protein	1.70	2.41
*ZMO1237*	Lactate dehydrogenase	1.70	2.19
*ZMO1289*	Putative transglycosylase-associated protein	2.06	3.23
*ZMO1522*	TonB-dependent receptor	2.75	3.88
**Down-regulated gene in ZM4 cultured in RM^OXOID^ compared with RM^BD^**			
*ZMO0934*	Secretion-related protein	**−**1.65	3.28
*ZMO0374*	Levansucrase	**−**1.45	3.34
*ZMO0383*	Hypothetical protein	**−**1.34	2.26
*ZMO0687*	Acetolactate synthase large subunit	**−**1.19	3.23
*ZMO0694*	Hypothetical protein	**−**1.08	3.38
*ZMO0930*	Hypothetical protein	**−**1.06	2.11
*ZMO0389*	Constituent protein	**−**1.04	2.07

*Ratio is the log_2_-based expression difference between ZM4 cultured in RM^OXOID^ and RM^BD^ (RM^OXOID^/RM^BD^).*

**TABLE 4:** List of significantly differentially expressed genes between ZM4 cultured in RM^OXOID^ and RM^OXOID^ + 8Mg.

**Name**	**Product**	**Ratio**	**−log_10_ (*P*-value)**
**Up-regulated gene in ZM4 cultured in RM^OXOID^ compared with RM+8Mg**			
*ZMO1522*	TonB-dependent receptor	1.47	2.88
*ZMO0286*	DUF541 domain-containing protein	1.31	2.40
*ZMO1754*	Succinate-semialdehyde dehydrogenase SSADH	1.21	2.47
*ZMO0918*	Catalase	1.18	2.49
*ZMO1289*	Putative transglycosylase-associated protein	1.17	2.35
*ZMO1776*	Aminopeptidase N	1.17	2.86
*ZMO1113*	NADH dehydrogenase	1.15	2.47
*ZMO0968*	Hypothetical protein	1.09	2.37
*ZMO0740*	General stress protein CsbD	1.03	2.05
*ZMO1285*	Sorbitol dehydrogenase large subunit	1.02	2.55
**Down-regulated gene in ZM4 cultured in RM^OXOID^ compared with RM+8Mg**			
*ZMO0066*	Hypothetical protein	**−**1.20	2.02
*ZMO0934*	Secretion-related protein	**−**1.26	2.85
*ZMO0374*	Levansucrase	**−**1.26	3.12
*ZMO0381*	Hypothetical protein	**−**1.40	2.56

**Ratio is the log_2_-based expression difference between ZM4 cultured in RM^*OXOID*^ and RM^*OXOID*^ + 8Mg (RM^*OXOID*^/RM^*OXOID*^ + 8Mg).**

Genes related to stress responses were up-regulated while genes related to protein secretion were down-regulated in RM^OXOID^ medium compared with in RM^OXOID^ + 8Mg or RM^BD^ medium (Figure 9 and Tables 3, 4). For example, genes encoding catalase (*ZMO0918*), general stress protein CsbD (*ZMO0740*), NADH dehydrogenase (*ZMO1113*), and succinate-semialdehyde dehydrogenase SSADH (*ZMO1754*) were up-regulated when RM^OXOID^ was used compared with those in RM^OXOID^ + 8Mg or RM^BD^ (Figure 9 and Tables 3, 4). These results indicate that Mg^2+^, a key element of cofactor, is essential for vigorous cell growth, and the lack of Mg^2+^ will trigger energy-consuming stress responses while slowing down energy-consuming metabolism with genes encoding levansucrase (*ZMO0374*) and secretion-related protein (*ZMO0934*) being down-regulated (Figure 9 and Tables 3, 4).

Considering that the supplementation of 8 mg/L Mg^2+^ into RM^OXOID^ (RM^OXOID^ + 8Mg) could restore the coupling of cell growth, glucose consumption and ethanol fermentation of *Z. mobilis* (Figure 8) and that there was only one gene up-regulated (*ZMO2034*) and one down-regulated (*ZMO1522*) when *Z. mobilis* grew in RM^BD^ compared with RM^OXOID^ + 8Mg (Figure 9C), the transcriptomics study further confirmed our hypothesis that the difference of Mg^2+^ concentrations in different nitrogen sources is one of the determinants affecting the coupling of cell growth, glucose consumption and ethanol fermentation in *Z. mobilis*.

## Conclusion

The effects of nitrogen sources on cell growth, glucose consumption, and ethanol fermentation performance of *Z. mobilis* ZM4 were investigated to understand the uncoupling of cell growth, glucose consumption and ethanol fermentation of ZM4 in this study. Through the supplementation and replacement of inorganic or organic nitrogen sources in both RM and MM, we found that YE^BD^, YE^SG^, or 5% CSL were better than YE^OXOID^. We also quantified the ion elements in different nitrogen sources using ICP-OES, and demonstrated that the difference of magnesium ion in YE is one of the major factors affecting cell growth and ethanol fermentation. We further verified the impact of Mg^2+^ on cell growth of ZM4 by supplementing various concentrations of Mg^2+^ into the medium, and used the RNA-Seq transcriptomics approach to identify genes responsive for the uncoupling of cell growth, glucose consumption and ethanol fermentation when the medium lacked Mg^2+^. These findings can be used as a reference for the selection and/or modification of nitrogen sources using *Z. mobilis* ZM4. The concentrations of ion elements in nitrogen sources affecting cell growth and fermentation performance can also be used as a parameter for optimizing and monitoring the components of nitrogen sources for efficient cell growth and fermentation using other microorganisms.

## Materials and Methods

### Bacterial Strain, Media, and Growth Conditions

*Zymomonas mobilis* ZM4 (ATCC 31821) was revived from frozen glycerol stocks in 10 mL RM^OXOID^ (50 g/L glucose, 10 g/L YE, 2 g/L KH_2_PO_4_) at 30°C for about 6∼8 h prior to inoculating overnight seed cultures at 30°C at 100 rpm in RM^OXOID^ using shake flasks with a sealing gas permeable membrane sealed filled to 80% capacity. Glucose, KH_2_PO_4_, K_2_HPO_4_, NaCl, MgSO_4_⋅7H_2_O, Na_2_MoO_4_⋅2H_2_O, and NH_4_Cl were purchased from Sinopharm Chemical Reagent Co., Ltd. (Shanghai, China). YE was purchased from OXOID, Becton Dickinson, and Sangon Biotech (Shanghai) Co., Ltd. (Shanghai, China). Tryptone was purchased from OXOID. Peptone was purchased from Becton Dickinson. Corn steep liquid (CSL) was purchased from Shanghai Macklin Biochemical Co., Ltd. (Shanghai, China).

The recipes of different rich medium (RM) and minimal medium (MM) with different nitrogen sources used in this work were listed in Tables 5, 6, respectively.

**TABLE 5:** Recipes of different rich medium (RM) with different nitrogen sources used in this work.

**Media**	**Nitrogen sources**
RM(P)	Peptone (10 g/L)
RM(T)	Tryptone (10 g/L)
RM(1%CSL)	CSL (10 g/L)
RM(5%CSL)	CSL (50 g/L)
RM^BD^	YE^BD^ (10 g/L)
RM^SG^	YE^SG^ (10 g/L)
RM^OXOID^	YE^OXOID^ (10 g/L)
RM + P	YE^OXOID^ (10 g/L) + peptone
RM + T	YE^OXOID^ (10 g/L) + tryptone

**All media contained ingredients of glucose (50 g/L), KH_2_PO_4_ (2 g/L), and nitrogen sources. YE*, *Yeast extract; CSL*, *corn steep liquid. The media supplemented with CSL were filter-sterilized, others were autoclaved (108°C, 30 min).**

**TABLE 6:** Recipes of different minimal medium (MM) with different nitrogen sources and metal ions of Mg^2+^ and MoO_4_^2^**^–^** used in this work.

**Media**	**Nitrogen sources**	**MgSO_4_⋅7H_2_O (0.5 g/L)**
MM	(NH_4_)_2_SO_4_ (1 g/L)	**+**
MM(5%CSL)	CSL (50 g/L)	**+**
MM^OXOID^	YE^OXOID^ (10 g/L)	**+**
MM^BD^	YE^BD^ (10 g/L)	**+**
MM^OXOID^ΔMgSO_4_	YE^OXOID^ (10 g/L)	**−**
MM^BD^ΔMgSO_4_	YE^BD^ (10 g/L)	**−**

**YE*, *Yeast extract; CSL*, *corn steep liquid. All media contained ingredients of glucose (20 g/L), K_2_HPO_4_ (1 g/L), KHP_2_O_4_ (1 g/L), NaCl (0.5 g/L), and nitrogen sources. The media supplemented with CSL were filter-sterilized, others were autoclaved (108°C, 30 min). After the autoclavation, the filter-sterilized Na_2_MoO_4_⋅2H_2_O (0.025 g/L) was then added into the media, and the filter-sterilized MgSO_4_⋅7H_2_O (0.5 g/L) was also added into the media as needed****+****: with the component in the medium;***−***: without the component in the medium.**

### Growth Test by Bioscreen C

The Bioscreen C automatic growth curve analyzer has the functions of culturing cells and measuring cell turbidity. It is similar to the plate reader, but can continuously measure the turbidity of cells, while maintaining a constant temperature and rotation speed to maintain normal cell growth.

The seed culture of *Z. mobilis* was centrifuged to remove RM^OXOID^. Cells were resuspended with test medium. Bioscreen C assays were carried out as described previously (Franden et al., 2009; Yang et al., 2010, 2014a,b, 2016b; Yang S. et al., 2018) except that cells were inoculated into Bioscreen C wells containing a total volume of 200 μL test medium at an initial OD_600_ value of 0.05 and incubated without shaking at 30°C. Triplicate were used for each condition, and turbidity measurements (OD_600_) were taken every 15 min till cells grew into stationary phase. At least two independent experiments were carried out with similar results. Values are the mean of one representative experiment with two or more technical replicates. Error bars represent standard deviations.

### Flask Fermentation and Analytical Analysis

The seed culture of *Z. mobilis* was used to inoculate the shake flask containing 80% of test medium with a sealing gas permeable membrane sealed at an initial OD_600_ of 0.1, and cultured at 30°C, 100 rpm. At least two replicates were used for each condition.

The OD_600_ values of the bacterial culture was measured by UV-visible spectrophotometer UV-1800 (AoYi Instrument Co., Ltd., Shanghai, China) every 3 h. At the same time, 1-mL culture was centrifuged at 12,000 rpm for 1 min to obtain the supernatant for measuring the glucose and ethanol concentrations in the culture. Biosensor analyzer M-100 (Sieman Technology Co., Ltd., Shenzhen, China) was used for quick assessment of the concentrations of glucose and ethanol. The supernatant was also filtered through a 0.45 μm filter before applying on a Shimadzu LC-2030 high pressure liquid chromatography (HPLC) with refractive index detector (RID). Bio-Rad Aminex HPX-87H (300 × 7.8 mm) column was used to separate the fermentation products, and 0.005 M H_2_SO_4_ was used as the mobile phase at a flow rate of 0.5 mL/min. Temperatures of detector and column were 40 and 60°C, respectively. The measurement of glucose and ethanol by HPLC and Biosensor analyzer M-100 was compared, and the results showed that the measurement data of the two instruments on the same sample were close (data not shown). At least two independent experiments were carried out with similar results. Values are the mean of one representative experiment with two or more technical replicates. Error bars represent standard deviations.

One percent (w/v) of different organic nitrogen sources were prepared in ddH_2_O and then filter-sterilized. The concentrations of different ions in these samples were then measured by Inductively Coupled Plasma Optical Emission Spectroscopy (ICP-OES, Wuhan SouSepad Testing Technology Co., Ltd., Wuhan, China).

### Ethanol Production Calculations

Cell growth was monitored by optical density spectrophoto- metrically at 600 nm. The time points when cells reached stationary phase and when glucose was completely consumed with the maximum ethanol produced were recorded. The concentrations of glucose and ethanol determined by HPLC or Biosensor analyzer M-100 were then used for the calculation of the ethanol yield (***Y*_*p/s*_**), theoretical ethanol yield **(*%Y_*p/s*_***), ethanol productivity (***Q*_*P*_**), and specific growth rate (**μ**) using the formula below:

***Y_*p/s*_*** = maximum ethanol (g)/consumed glucose (g);

***%Y_*p/s*_*** = (ethanol(g)/(consumed sugar(g)*0.511))*100;

***Q_*P*_*** = maximum ethanol titer (g/L)/time to reach the maximum ethanol.

**μ** = ln((OD_600_ at t_2_)/(OD_600_ at t_1_))/(t_2_−t_1_), t_1_ and t_2_ are the time points in the log phase.

### Transcriptomic Analysis

The transcriptomics study followed previous work (Franden et al., 2009; Zhao et al., 2009; Martinez-Moreno et al., 2012; Kemsawasd et al., 2015; Klotz et al., 2017; Yang S. et al., 2018; Lozano Terol et al., 2019). Briefly, cell culture samples were collected during the exponential phase with the OD_600_ values around 0.6–0.8. RNA-Seq was performed using paired-end sequencing technology according to standard Illumina protocols (Genewiz Inc., Suzhou, China). The quality of RNA-Seq fastq data was evaluated using FastQC software (Babraham Bioinformatics, United States). Data passing the quality control were imported into CLC Genomics Workbench (Ver. 11.0) for RNA-Seq analysis to get the RPKM value (reads mapping to the genome per kilobase of transcript per million reads sequenced) of each gene with the reference genome. Genome sequence of *Z. mobilis* was used as the reference for RPKM calculation (Yang et al., 2010; Martinez-Moreno et al., 2012). The RPKM value of each gene was then imported into JMP Genomics (Ver. 9.0); data normalization and statistical analysis were conducted to identify differentially expressed genes when three different media were used. Duplicate samples were used for each condition. RNA-Seq raw data were deposited at Sequence Read Archive (SRA) database with the Bioproject accession number of PRJNA601020.

## Data Availability Statement

The RNA-Seq raw data was deposited at Sequence Read Archive (SRA) database with the BioProject accession number PRJNA601020.

## Author Contributions

SY conceived and designed the experiments with inputs from RL, MJ, SC, ML, and JD. RL performed the experiments. RL and SY wrote the manuscript. MJ, ML, SC, and JD conducted the extensive manuscript review. All authors contributed to the article and approved the submitted version.

## Conflict of Interest

JD was employed by China Biotech Fermentation Industry Association. ML was employed by Zhejiang Huakang Pharmaceutical Co., Ltd.

The remaining authors declare that the research was conducted in the absence of any commercial or financial relationships that could be construed as a potential conflict of interest.

The reviewer QG declared a past co-authorship with one of the authors SY to the handling editor.

## Abbreviations

CSL: corn steep liquid
ED: Entner-Doudoroff
EMP: Embden-Meyerhof-Parnas
GRAS: generally regarded as safe status
MM: minimum medium
ICP-OES: Inductively Coupled Plasma Optical Emission Spectroscopy
P: peptone
RM: rich medium
RM^BD^: yeast extract in rich medium was from company Becton Dickinson (BD)
RM^OXOID^: yeast extract in rich medium was from company OXOID
RPKM: reads mapping to the genome per kilobase of transcript per million reads sequenced
T: tryptone
TRY: titer, rate and yield
YE: yeast extract.
